# Editorial: Integrated biosensors towards clinical and point-of-care diagnostics

**DOI:** 10.3389/fbioe.2022.1008603

**Published:** 2022-09-12

**Authors:** Can Dincer, Pranjal Chandra, Eden Morales-Narváez

**Affiliations:** ^1^ FIT Freiburg Center for Interactive Materials and Bioinspired Technologies, University of Freiburg, Freiburg, Germany; ^2^ Department of Microsystems Engineering (IMTEK), University of Freiburg, Freiburg, Germany; ^3^ Laboratory of Bio-physio Sensors and Nanobioengineering, School of Biochemical Engineering, Indian Institute of Technology (BHU), Varanasi, Uttar Pradesh, India; ^4^ Biophotonic Nanosensors Laboratory, Centro de Investigaciones en Óptica León, Guanajuato, Mexico

**Keywords:** health 4.0, on-site detection, sample preparation, microfluidics, non-invasive diagnostics, point-of-care testing

Biosensing devices such as lateral flow assays, blood glucose test strips or wearable continuous glucose monitoring systems have had a great impact on healthcare for more than half a century and have already improved our living standards. In particular, biosensors allow us to measure clinically relevant biomarkers from various sample matrices (including whole blood, plasma, saliva, or urine) in a fast, cost-effective, and user-friendly manner. Hence, biosensing technologies can provide vital information related to the propensity for illness or the status of a disease, or its treatment monitoring. However, there are still some challenges, especially regarding the sample Research Topic and preparation as well as the biosensing performance of diagnostic devices (Ates et al., 2021).

To further assess the potential of this technology, researchers are pushing forward the frontiers of integrated biosensors, combining the sample Research Topic, preparation, analysis, and data evaluation, to create “sample-in-answer-out” diagnostic devices. Such integrated solutions for biosensing could enable real-time, highly sensitive, and selective quantification of various biomolecules within clinically relevant body fluids in a smart, sustainable, interoperable, and portable manner.

This Research Topic gathers innovative approaches for integrated biosensing systems conceived from colleagues located in diverse parts of the world, including Australia, China, Denmark, Taiwan, the United Kingdom, and the United States. Several clinically relevant agents are targeted, including respiratory viruses (Fan et al.), interleukin-6 (a biomarker of inflammatory associated diseases) (Rahbar et al.), biomarkers related to gastric cancer (Liu et al.), dengue serotypes (Moser et al.), SARS-CoV-2 virus (Lin et al.; Ngoc et al.), and carbohydrate antigen 50 (a non–organ-specific tumor biomarker for screening several cancers) (Liu et al.). All of them were presented with (potential) clinical applications at the point-of-care. In particular:

Fan et al. integrated recombinase aided amplification (a novel isothermal amplification method targeting the genetic material of various pathogens) and quantitative polymerase chain reaction (qPCR) in order to detect respiratory viruses (ADV3 and ADV7). A conventional qPCR apparatus was employed to perform nucleic acid amplification and highly specific detection in a single tube within 1 h.

Rahbar et al. reported a paper-based device where the silver amplification technique was integrated in a lateral flow immunoassay to enhance colorimetric signals, leading to a reliable, low-cost, and user-friendly platform for the detection of interleukin-6.

Liu et al. reviewed the state of the art of Raman spectroscopy as an emerging label-free technology for gastric cancer diagnosis. The authors highlight that the integration of a Raman fiber optic probe and endoscope facilitates real-time *in vivo* detection of cancer development. In this context, limiting factors to be addressed are also spotted, including those related to equipment, clinical sensitivity/specificity, and Raman data analysis.

Lin et al. integrated a molecular diagnostic device with microfluidic chips and dual temperature modules. This platform offers detection of the genetic material of pathogens in less than 24 min. The clinical sensitivity of the platform was demonstrated by analyzing clinical samples of sputum and stool, thereby enabling a rapid, reliable, and user-friendly device for the detection of pathogens such as SARS-CoV-2.

Liu et al. implemented a miniaturized chemiluminescence immunoassay analyzer that obviates the skills of particularly trained personnel. For the demonstration of the proof-of-concept, carbohydrate antigen 50 was detected using this platform. The analyzer, which can detect 60 samples per hour, was proven technically sound with clinical samples, offering an alternative to performing cancer-related diagnostics in low-resource settings.

Ngoc et al. developed a point-of-care system for the fast and real-time detection of SARS-CoV-2. The team took advantage of fluorescent signals and Arduino platforms (compatible with commercially available open-source hardware-software and off-the-shelf electronic components) to control real-time reverse transcriptase loop-mediated isothermal amplification reactions for the quantitative detection of SARS-CoV-2 viruses.

Moser et al. introduced a smartphone-connected portable lab-on-a-chip platform for the quantitative detection of two dengue serotypes. A smartphone app synchronizes the obtained results in real-time with a secure cloud server hosted by Amazon Web Services for epidemiological surveillance. In addition, the portable character and geo-tagging abilities of the platform were proven to be technically sound in low-resource settings employing a pilot study performed between United Kingdom and Taiwan.

We hope this article Research Topic contributes valuable insights into the exciting field of integrated biosensors with comprehensive knowledge of fundamentals, materials, technologies, and their point-of-care applications. This in turn will help researchers to conduct research beyond the state-of-the-art in this field.
